# Draft Genome Sequence of Actinobacterial Strain Kineosporia sp. R_H_3, a Neutrophilic Iron-Depositing Bacterium

**DOI:** 10.1128/genomeA.00335-18

**Published:** 2018-04-12

**Authors:** Burga Braun, Sven Künzel, Josephin Schröder, Ulrich Szewzyk

**Affiliations:** a Technische Universität Berlin, Berlin, Germany; b Max Planck Institute for Evolutionary Biology, Plön, Germany

## Abstract

The draft genome sequence of a neutrophilic iron-depositing actinobacterial strain, Kineosporia sp. R_H_3, is reported here. Detailed analysis of the genome can elucidate the role of specific cytochromes for Fe oxidation and how this organism might receive energy from Fe oxidation. To date, this is the second publicly available genome sequence of a Kineosporia strain.

## GENOME ANNOUNCEMENT

Kineosporia sp. strain R_H_3 is a small Gram-positive bacterium that formed very small dark-brown colonies with a diameter of less than 1 mm on agar plates containing iron and manganese. BLAST searches of a 1,504-bp sequence of the 16S RNA gene using the Nucleotide Collection Nr/Nt database (1) and EzBioCloud (2) revealed five of the seven described Kineosporia strains as the closest relatives, namely, strains NBRC 16234^T^, JCM 9954^T^, VN05A0415^T^, JCM 9957^T^, and YIM 65293^T^, each with an identity of 96%.

Strain R_H_3 was isolated from ochrous formations of a 320-m-deep dewatering well of the open cast mining area in Hambach, Germany (Rhenish lignite-mining area). For isolation, diluted ochrous samples were spread on H_2_O medium [2 g liter^−1^ MnCO_3_ hydrate, 0.2 g liter^−1^ Fe(NH_4_)_2_(SO_4_)_2_, 2 ml vitamin solution (3), 2 ml trace element solution SL 9 (4), 15 g agar, and 1 liter deionized water] and incubated at room temperature until dark-brown colonies developed. The iron deposition ability of the strain was verified according to Braun et al. (5). Determination of the physiological characteristics using Biolog GN2 microplates (I&L Biosystems GmbH, Königswinter, Germany) showed that strain R_H_3 was able to metabolize only Tween 40 and Tween 80. pH-dependent growth rates were determined in R2A medium (DSMZ 830), and optimal growth occurred at pH 6. On agar plates, strain R_H_3 was able to grow on H_2_O medium and on LSM2 (6), CY (DSMZ 67), and R2A and NA (DSMZ 1) media.

Extraction of genomic DNA was done as previously described (7), and a paired-end library was prepared according to the Illumina Nextera XT DNA library prep kit protocol. Genome sequencing was performed on an Illumina NextSeq 500 sequencer using NextSeq mid output kit v2 (300-cycle) chemistry, which generated 19,900,890 raw reads. Demultiplexing was done with bcl2fastq v2.18.0.12, and quality filtering of raw reads was performed using Trimmomatic v0.36 (8), resulting in 13,319,870 filtered reads. Subsequently, reads were checked for ambiguous base calls and low complexity employing the DUST algorithm (9) and filtered accordingly with an R script in Microsoft R Open v3.3.2 (http://www.r-project.org/), followed by preassembly with SPAdes v3.10.0 (10) using default k-mer lengths up to 99 bp. Scaffolds of ≥500 bp of this preassembly were subject to extension and second-round scaffolding with SSPACE standard v3.0 (11). Scaffolds of ≥2,500 bp were assigned to genome bins by MetaBAT v0.32.4 (12), and functional annotation of the draft genomes was performed with Prokka v1.12b (13). The draft genome included 270 scaffolds with an *N*_50_ assembly quality of 39,507 and an *L*_50_ value of 53. The shortest scaffold was 2,548 bp, and the longest scaffold was 206,123 bp. The total size of the scaffolds was 7,260,683 bp, with a GC content of 74%. Annotation resulted in 6,590 coding regions for 6,678 genes, 514 signal peptides, no clustered regularly interspaced short palindromic repeat (CRISPR) repeat units, 3 rRNAs (5S, 16S, and 23S), 68 tRNAs, 1 transfer-messenger RNA (tmRNA), and 16 miscellaneous RNAs.

### Accession number(s).

This whole-genome shotgun project has been deposited at DDBJ/ENA/GenBank under the accession number MWLL00000000. The version described in this paper is version MWLL01000000.
